# Increased ex vivo cell death of central memory CD4 T cells in treated HIV infected individuals with unsatisfactory immune recovery

**DOI:** 10.1186/s12967-015-0601-2

**Published:** 2015-07-17

**Authors:** Marta Massanella, Elisabet Gómez-Mora, Jorge Carrillo, Marta Curriu, Dan Ouchi, Jordi Puig, Eugènia Negredo, Cecilia Cabrera, Bonaventura Clotet, Julià Blanco

**Affiliations:** Institut de Recerca de la Sida IrsiCaixa-HIVACAT, Institut d’Investigació en Ciències de la Salut Germans Trias i Pujol, Universitat Autònoma de Barcelona, 08916 Badalona, Spain; Fundació Lluita contra la SIDA, Institut d’Investigació en Ciències de la Salut Germans Trias i Pujol, 08916 Badalona, Spain; Universitat de Vic-Central de Catalunya, UVIC-UCC, 08500 Vic, Spain; Department of Pathology, University of California San Diego, La Jolla, CA 92093 USA

**Keywords:** Immunodiscordant, Cell death, T-cell subsets, Immunosenescence, HAART, cART

## Abstract

**Background:**

High levels of ex vivo CD4 T-cell death and the accumulation of highly differentiated and/or immunosenescent T cells have been associated with poor CD4 T-cell recovery in treated HIV-infected individuals. However, the relationship between cell death and T-cell differentiation is still unclear.

**Methods:**

We have analyzed cell death, immunosenescence and differentiation parameters in HAART-treated subjects (VL <50 copies/mL for more than 2 years) with CD4 T-cell count <350 cells/μL (immunodiscordant, n = 23) or >400 cells/μL (immunoconcordant, n = 33). We included 11 healthy individuals as reference.

**Results:**

As expected, suboptimal CD4 T-cell recovery was associated with low frequencies of naïve cells, high frequencies of transitional and effector memory cells and a subsequent low ratio of central/transitional memory cells in the CD4 compartment. These alterations correlated with spontaneous CD4 T-cell death. A deeper analysis of cell death in CD4 T-cell subsets showed increased cell death in memory cells of immunodiscordant individuals, mainly affecting central memory cells. Immunosenescence was also higher in immunodiscordant individuals albeit unrelated to cell death. The CD8 compartment was similar in both HIV-infected groups, except for an underrepresentation of naïve cells in immunodiscordant individuals.

**Conclusion:**

Immunodiscordant individuals show alterations in memory CD4 T-cell differentiation associated with a short ex vivo lifespan of central memory cells and an in vivo low central/transitional memory cell ratio. These alterations may contribute to poor CD4 T-cell repopulation.

**Electronic supplementary material:**

The online version of this article (doi:10.1186/s12967-015-0601-2) contains supplementary material, which is available to authorized users.

## Background

HIV-1 infection leads to major perturbations of immune regulatory mechanisms [1] as a consequence of virus-mediated depletion of CD4 T cells [2, 3] and increased immune activation and inflammation [1]. Abnormal proliferative responses, maturation and immunosenescence of T cells, affecting both CD4 and CD8 compartments and covering acute, chronic and late stages of infection have been reported [4].

Highly active antiretroviral therapy (HAART) efficiently controls HIV-1 replication, but inflammatory status and immune activation remain abnormally high in treated individuals compared with uninfected individuals [1]. Furthermore, CD4 T-cell recovery shows a heterogeneous response [5, 6]; while some subjects reach CD4 T-cell counts comparable to healthy individuals, others fail to increase CD4 T-cell counts even after long-term (>5 years) virologically effective treatment [5, 7]. This immunodiscordant response has been associated with immunological damage induced by HIV-1 replication measured by nadir CD4 T-cell counts [7, 8] and specifically with the destruction of thymic tissue and the consequent lack of production of new naïve cells [9, 10]. However, in the absence of new T cells, memory cells can repopulate the CD4 T-cell compartment in non-human primate models of HIV infection [11], suggesting that the damage of memory cells may also contribute to blunted immunological recoveries.

Increased replicative senescence, measured by the senescence marker CD57 [12], have been related with immune recovery [13] and could affect CD4 T cells in both naïve and memory subsets [14]. Apart from replicative senescence, skewed maturation of memory CD4 T cells [15] and higher turnover and sensitivity to spontaneous cell death [9, 15] may contribute to a lower proliferative potential of memory cells leading to incomplete immune recovery under HAART. However, it is still unclear how these different alterations of memory cells are linked and how are they associated with thymic damage or senescence. Moreover, the specific role of central memory CD4 T cells, a major player of HIV pathogenesis [16], in immune recovery remains poorly defined.

Considering the role of CD4 T-cell death in immunological recovery on HAART [6], we sought to analyze its relationship with altered maturation/immunosenescence profiles of memory cells. Therefore, we designed a study to compare these parameters in subjects with low and high CD4 T-cell recovery after long-term successful HAART. An aberrant maturation phenotype defined by a reduced central/transitional memory CD4 T-cell ratio, but not immunosenescence, strongly correlated with poor CD4 T-cell recovery and seems to be associated with high cell death levels in intermediate differentiation stages of memory cells.

## Methods

### Samples

A cross-sectional study was performed to analyze the level of immunosenescence and differentiation of T cells in HAART-treated subjects. The Institutional Review Board of the Hospital Germans Trias i Pujol approved the study (EO code: EO-07-024) and all individuals provided written informed consent. Subjects that participated in the APOP-V^+^I^−^ study [8, 17] were screened for the current study: 56 individuals with at least two frozen aliquots of PBMCs were selected. All subjects had confirmed diagnosis of HIV infection, continuous HAART with plasma viral load <50 copies/mL for at least the last 2 years (minimum of 4 determinations during this period) and good treatment adherence. Exclusion criteria were: chemotherapy or interferon/ribavirin treatment and history of opportunistic infections during the last 2 years.

Patients were classified as Concordant (favorable virologic and immunologic response) when CD4 T-cell counts were above 400 cells/μL. Conversely, Discordant patients (favorable virologic but unsatisfactory immunologic response) showed CD4 T cells <350 cells/μL [8, 17]. Clinical and demographic data were collected from medical records. For comparative purposes, frozen PBMC from a control group of HIV uninfected individuals (n = 11; EO-10-007, Institutional Review Board of the Hospital Germans Trias i Pujol) were also analyzed.

### CD4 and CD8 T-cell immunophenotype

Frozen PBMCs were thawed at 37°C, washed twice in RPMI/20% of fetal bovine serum (FBS) and incubated for 1 h at 37°C before staining with a previously described eight-color panel [18] including CD3-APC-Cy7, CD4-PerCP-Cy5.5, CD8-V500, CD57-FITC, CD27-APC, CD28-PE, CCR7-PE-Cy7 and CD45RA-V450 (BD Biosciences). Cell viability was evaluated by Propidium Iodide (PI, Sigma-Aldrich) staining in parallel tubes, the median [IQR] of PI^+^ cells was 2.1% [1.5–4.0]. Lymphocyte gate was defined by morphological parameters excluding most dead cells (less than 0.1% of PI^+^ cells were found in the living lymphocyte gate). At least 300,000 lymphocytes were acquired in a LSRII cytometer (BD Biosciences) and analyzed with FlowJo software (Tree Star). Gating strategy is described in Additional file 1: Figure S1. Replicative senescence was defined by CD57^+^CD28^−^ cells. T-cell subsets were first analyzed for CD27 and CD28 expression to identify Terminally Differentiated cells (T_TD_, CD28^−^CD27^−^) and Effector Memory cells (TEM, CD27^−^CD28^+^ for CD4 T cells or CD27^+^CD28^−^ for CD8 T cells). Double positive CD27^+^CD28^+^ cells were subanalyzed for CD45RA and CCR7 expression allowing for the definition of naive (T_N_, CD45RA^+^CCR7^+^), central memory (T_CM_, CD45RA^−^CCR7^+^) and transitional memory cells (T_TM_, CD45RA^−^CCR7^−^). For each subset the expression of CD57 was analyzed (Additional file 1: Figure S1).

Immune activation, absolute lymphocyte counts (used to calculate absolute counts of each subset) and thymic production (CD45RA^+^CD31^+^CD4 T cells) were previously assessed in fresh blood samples in both CD4 and CD8 T cells [8, 17].

### Ex vivo culture of whole PBMC and sorted cells

Data on spontaneous CD4 and CD8 T-cell death (including necrosis, intrinsic and extrinsic apoptosis) have been previously reported and were obtained after culture of freshly obtained PBMC for 24 h [17, 19]. Furthermore, in a subgroup of patients (n = 10) freshly obtained PBMC were stained with CD3-APC-Cy7, CD4-PerCP-Cy5.5, CD8-V500, CD27-APC, CCR7-PE-Cy7 and CD45RA-V450 as described above, washed in PBS and sorted immediately in a FACSAria (BD Biosciences) to obtain gated CD3^+^CD4^+^CD8^−^ cells with the following phenotypes: T_N_ cells (CD27^+^CD45RA^+^CCR7^+^), T_CM_ cells (CD27^+^CD45RA^−^CCR7^+^), T_TM_ cells (CD27^+^CD45RA^−^CCR7^−^) and a mixture of T_EM_ and T_TD_ cells (T_EM+TD_ CD27^–^). These latter subsets were sorted as a single population due to technical limitations of sorting procedure (only 4 sorted populations could be obtained from a single sample). Sorted cells were cultured for 24 h and cell death was assayed using 40 nM DIOC(6) as described previously [19]. DIOC(6)^low^ cells, having lost mitochondrial membrane potential, were identified as dead cells.

### Statistical analysis

Continuous variables were expressed as the median (interquartile range) and compared using Mann–Whitney U test, permutation test for unbalanced groups or Signed-Rank test (for paired analyses). Discrete variables were described as percentages and compared using the Fisher´s exact test. Correlations were assessed by the Spearman’s test. Multiple comparisons were adjusted for false discovery rate. Statistical analyses were performed using R software version 3.0.2 [20] with two-tailed significance levels of 5%.

## Results

### Subject characteristics

**Figure 1:**
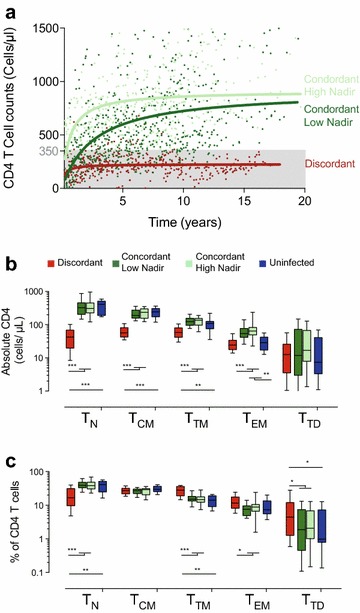
Characterization of CD4 T-cell subsets. **a** shows the evolution of CD4 T-cell counts in the three groups defined in this study (Immunodiscordant in *red* (n = 23), immunoconcordant with low and high nadir in *dark* and *light green*, m = 17 and n = 16, respectively). *Dots* correspond to individual determinations of CD4 T-cell counts and* lines* show non-lineal regression of data plotted for comparative purposes. **b** The absolute count of circulating and T_N_, T_CM_, T_TM_, T_EM_ and T_TD_ CD4 T cells was analyzed in immunodiscordant individuals (*red boxes*), immunoconcordant individuals with low or high nadir (LN and HN; *dark* and *light green boxes*, respectively) and healthy HIV uninfected individuals (n = 11, *blue boxes*). **c** The frequencies of the above-mentioned subsets in the CD4 T-cell compartment were also analyzed. In* panels* **b** and **c**, data shown correspond to median values (*band*), IQR (*boxes*) and 10–90 interquartile values (*whiskers*). Figure show significant *p* *values* (permutation test adjusted by false discovery rate): *p < 0.05; **p < 0.01 and ***p < 0.005.

Subjects recruited in this study were stratified according to the level of recovery of CD4 T cells after suppressive HAART, as immunodiscordant or immunoconcordant (cutoff value 350 CD4 T cells/μL). The time course of CD4 T-cell recovery for each group is shown in Figure 1a. At the time of sample analysis, the median CD4 T-cell count was 220 and 798 cells/μL for immunodiscordant and immunoconcordant HIV-infected individuals respectively (*p* < 0.0001), reflecting the blunted dynamics of CD4 T-cell recovery of the former group. Absolute CD8 T-cell counts showed no differences between HIV-infected groups. Median length of HIV infection (from diagnosis) was 11.8 and 10.1 years (*p* = ns), while median time on treatment was 11.2 and 5.2 years (*p* = ns) for immunoconcordant or immunodiscordant subjects, respectively. Overall, immunodiscordant individuals showed the previously reported higher CD4 T-cell death and activation with significantly lower nadir CD4 T-cell counts [8, 17]. However, some immunoconcordant individuals showed also low nadir values. To evaluate the effect of nadir on immune recovery, immunoconcordant individuals were further divided according to the median nadir CD4 T-cell value (cut-off value 250 CD4 T cells/μL) into two subgroups with low and high nadir values (n = 17 ad n = 16, respectively). This allowed the direct comparison of immunoconcordant individuals with immunodiscordant subjects, avoiding the confounding effect of nadir and enabling us to evaluate the impact of low nadir values in immunoconcordant individuals. No major differences were observed among immunoconcordant subgroups, except for a higher presence of PI-based treatments and HCV co-infection in the low nadir group (Table 1).

**Table 1:** Main characteristics of the different groups

	Discordant (n = 23)	a	Concordant			b	Uninfected (n = 11)
			All (n = 33)	Low Nadir (n = 17)	High Nadir (n = 16)		
Age (years), Median [IQR]	48 [45–50]	ns	45 [38–49]	48 [42–52]	42 [37–45]	ns	38 [34–47]
Gender (% of male)	91	ns	85	76	94	ns	55
Time since HIV diagnosis (years), Median [IQR]	10.1 [4.1–20.4]	ns	11.8 [7.5–16.6]	12.2 [8.9–17.4]	11.6 [4.9–13.4]	ns	–
Time on HAART (years), Median [IQR]	5.2 [3.5–11.4]	ns	11.2 [7.4–12.6]	11.4 [8.8–12.6]	10.6 [3.7–12.8]	ns	–
Current HAART (% PI-based)	70	*	33	41	25	*	–
HCV coinfection (%)	35	ns	21	36	15	*	0
Ratio CD4/CD8, Median [IQR]	0.23 [0.17–0.33]	*	0.87 [0.60–1.11]	0.76 [0.54–0.87]	1.06 [0.86–1.1]	ns	1.64 [1.31–1.81]
CD4 T cell counts (cells/µL), Median [IQR]	220 [192–253]	*	798 [600–998]	703 [600–896]	881 [672–1,075]	ns	779 [629–1,072]
Nadir (cells/µL), Median [IQR]	64 [15–122]	*	239 [76–345]	76 [19–185]	351 [280–429]	*	–
CD4 T-cell gain (cell/µL/year HAART), Median [IQR]	27 [9–54]	*	53 [46–102]	53 [46–93]	53 [36–124]	ns	–
CD4 T cell (% of lymph), Median [IQR]	14 [10–17]	*	31 [27–39]	29 [27–33]	37 [29–40]	ns	42 [36–45]
CD4 T-cell death (%), Median [IQR]	9.3 [7.5–15.1]	*	4.6 [3.3–5.9]	4.9 [4.4–5.6]	4.3 [2.9–6.1]	ns	4.0 [3.2–4.7]
CD38^+^CD45RA^−^ (% of CD4 T cells), Median [IQR]	37 [29–41]	*	25.7 [20–32]	26.5 [20–32]	25.1 [20–31]	ns	
HLA-DR^+^CD95^+^ (% of CD4 T cells), Median [IQR]	16 [7.7–21.6]	*	4.5 [3.7–6.7]	4.7 [4.2–6.3]	4.4 [3.2–7.1]	ns	2.0 [1.5–2.7]
CD8 T cell counts (cells/µL), Median [IQR]	940 [754–1,146]	ns	908 [771–1,239]	1,118 [855–1,380]	811 [637–1,121]	ns	459 [433–548]
CD8 T cell (% of lymph), Median [IQR]	56 [51–61]	*	38 [34–46]	39 [36–47]	36 [32–40]	ns	24 [23–27]
CD8 T cell death (%), Median [IQR]	7.1 [4.8–10.3]	ns	6.5 [5.0–12.4]	8.8 [6.1–11.0]	6.1 [4.8–14.9]	ns	3.9 [3.4–4.9]
CD38^+^CD45RA^−^ (% of CD8 T cells), Median [IQR]	31 [23–36]	*	23 [19–35]	25 [21–36]	21 [19–26]	ns	9 [5–13]
HLA-DR^+^CD95^+^ (% of CD8 T cells), Median [IQR]	12.6 [6.9–22.4]	ns	9.1 [5.6–13.0]	9.8 [5.4–13.6]	8.5 [5.6–13.0]	ns	
sCD14 (µg/mL), Median [IQR]	8.4 [7.7–10.2]	ns	8.8 [7.2–9.7]	9.2 [7.6–10.1]	8.0 [7.1–9.2]	ns	4.2 [3.9–4.6]

*a* Comparison of concordant and discordant subjects. * denotes *p* < 0.05; *ns* non significant (Mann–Withney U or Fisher exact test).

*b* Comparison of concordant subjects with low and high nadir. * denotes p < 0.05; *ns* non significant (Mann–Withney U or Fisher exact test).

### Analysis of the CD4 T-cell maturation

Absolute counts and frequency of different CD4 T-cell subsets were analyzed in immunodiscordant, immunoconcordant (low and high-nadir subgroups) and 11 uninfected individuals. The data show that lower CD4 T-cell counts in immunodiscordant subjects (Figure 1a) were the consequence of lower levels of T_N_, T_CM_, T_TM_ and T_EM_ cells compared with immunoconcordant individuals, while the absolute numbers of TTD cells were similar in all groups (Figure 1b). Interestingly, immunoconcordant patients, irrespective of the nadir values showed similar counts of all subsets that were in turn comparable to uninfected controls except for the T_EM_ subset, suggesting a proper recovery of the CD4 T-cell subsets in these individuals (Figure 1b). The frequency of each subset in the CD4 T-cell compartment showed a significant underrepresentation of TN cells in immunodiscordant subjects (as compared to concordant or HIV-uninfected individuals) that was compensated by an overrepresentation of T_TM_ cells and a less evident but still significant increase in T_EM_ and T_TD_ cells (Figure 1c). Conversely, T_CM_ cells showed similar values in all groups. Again, both subgroups of immunoconcordant subjects showed similar values of subset frequencies reaching the levels of HIV-uninfected controls (Figure 1c).

### CD4 T-cell maturation and CD4 T-cell death

**Figure 2:**
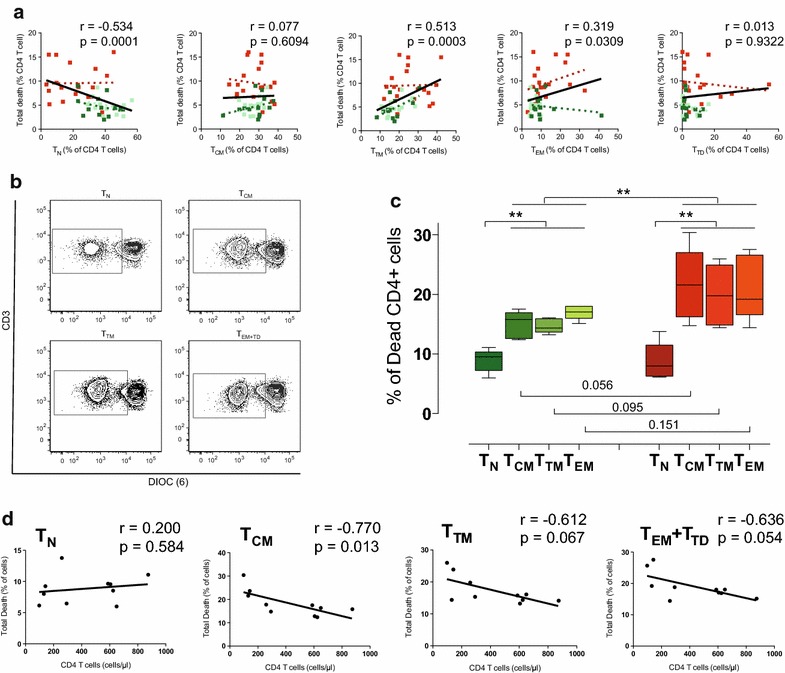
Association of CD4 T-cell maturation with CD4 T-cell death. **a** Relationships between the frequencies of the different CD4 T-cell subsets was plotted against spontaneous intrinsic CD4 T-cell apoptosis. Data from immunodiscordant (n = 23, *red*), low nadir immunoconcordant (n = 17, *dark green*) or high nadir immunoconcordant individuals (n = 16, *light green*) are shown with color-coded linear regression for each data set. Linear regression for the global data is shown by *black lines*. Correlation coefficients and *p* *values* of Spearman’s test for the global analysis are shown in each plot. **b** Spontaneous cell death was assessed in sorted T_N_, T_CM_, T_TM_, T_EM_/T_TD_ CD4 T cells stained with the potentiometric probe DIOC(6). Dot plots of DIOC(6) and CD3 staining for a representative individual show the percentage of dead cells in the *left* (DIOC low) gate. **c** The level of spontaneous cell death in sorted T_N_, T_CM_, T_TM_, T_EM+TD_ T cells from immunoconcordant (*green*, n = 5) or immunodiscordant (*red*, n = 5) treated HIV infected individuals are shown. Figure shows median values (*bands*), IQR (*boxes*) and 10-90 interquartile values (*whiskers*). *Asterisks* denote significant differences (non parametric permutation or Mann–Whitney tests). **d** Correlations of cell death sorted T_N_, T_CM_, T_TM_ and T_EM_/T_TD_ CD4 T cells with absolute counts of circulating CD4 T cells. Correlation coefficient and p values (Spearman) are shown in each graph.

In our previous studies we have shown that CD4 T-cell death, in particular intrinsic apoptosis, is a major determinant of immune recovery [8, 17]. Therefore, we explored the association of unbalanced CD4 T-cell maturation with the rate of cell death in ex vivo cultures of fresh PBMC. Spontaneous CD4 T-cell death was unrelated to the frequency of CD4 T_CM_ or T_TD_ cells but showed a significant negative correlation with the frequency of CD4 TN and positive correlation with T_TM_ and T_EM_ cells (Figure 2a). Since the frequency of CD4 T_N_ and T_TM_ cells were strongly inversely correlated (data not shown), we addressed independent associations by using a model including data from all subsets. This model (Additional file 2: Table S1) confirmed the independent positive association of CD4 T-cell death with the frequency of T_TM_ CD4 T cells, clearly linking the higher presence of these cells with the increased cell death observed in immunodiscordant individuals.

However, these data do not discriminate whether TTM cell death rate is similar among treated HIV infected individuals or is higher in immunodiscordant patients. To experimentally confirm these possibilities, we addressed the analysis of the lifespan of T_N_, T_CM_, T_TM_ and T_EM+TD_ CD4 T-cells from immunoconcordant and immunodiscordant individuals. Since PBMC culture did not allow for a direct assessment of cell death occurring in different CD4 T-cell subsets due to phenotypic changes upon cell death [21], we chose a previously reported sorting strategy prior to ex vivo culture to approach this issue [21]. Although this strategy may induce some additional stress to sorted cells, it allowed for a consistent measure of subset cell death (Figure 2b). In this set of experiments, we observed that TN cells showed significantly lower cell death rates than those observed in purified memory cells in both groups (Figure 2c). No significant difference in TN cell death was observed between groups. However, memory cells in immunodiscordant individuals showed higher rates of death (Figure 2c). This was associated with higher sensitivity to cell death in TCM cells from immunodiscordant individuals (*p* = 0.056), and non-significant trends in TTM and TEM_+_TD cells. Consistently, T_N_ cell death did not correlate with CD4 T-cell counts while a significant negative association (p = 0.013) was observed for T_CM_ and trends were noticed for T_TM_ and T_EM+TD_ cell death (p = 0.067 and 0.054, respectively; Figure 2d). No significant correlations with senescence markers could be identified (data not shown).

**Figure 3:**
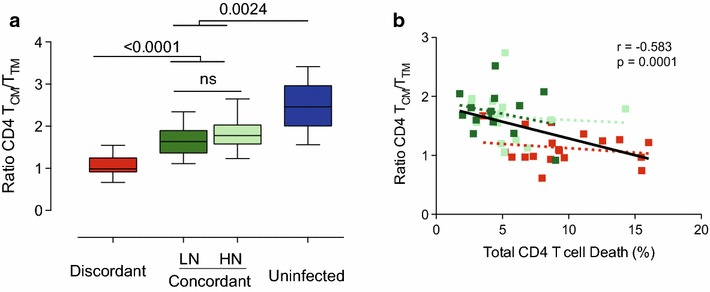
Ratio CD4 TCM/TTM and its association with total CD4 T-cell death. **a** The ratio between T_CM_ and T_TM_ cells was calculated as a measure of CD4 T-cell differentiation. Data shown correspond to median values (bands), IQR (*boxes*) and 10–90 interquartile values (*whiskers*) for immunodiscordant individuals (n = 23, *red boxes*), immunoconcordant individuals with low or high nadir (n = 17 and n = 16, *dark* and *light green boxes*, respectively) and healthy HIV uninfected individuals (n = 11, *blue boxes*). Figure shows significant *p* *values* (permutation test adjusted by false discovery rate). **b** Correlation between total CD4 T-cell death and the ratio of CD4 T_CM_/T_TM_ cells. *P* values for Spearman’s test correlation are shown in* black *for all data points, and in* red* or* green* for immunodiscordant and immunoconcordant individuals respectively.

To evaluate the impact of higher cell death rates on transition from T_CM_ to more advanced stages of CD4 T-cell maturation, we analyzed the ratio between T_CM_ and T_TM_ cells. This parameter was significantly lower in immunodiscordant subjects compared to immunoconcordant (irrespective of nadir values) or uninfected individuals (Figure 3a). Interestingly, T_CM_ to T_TM_ transition is not completely normalized in immunoconcordant individuals (Figure 3a), suggesting that this parameter could be useful to assess the quality of immune recovery. Reinforcing the relevance of the T_CM_/T_TM_ ratio, a strong negative correlation was observed between this parameter and spontaneous CD4 T-cell death (Figure 3b). Overall, our data suggest that memory, but not T_N_ cells, are major contributors to the increased cell death observed in immunodiscordant individuals [17]. Higher sensitivity to death of memory cell populations, especially T_CM_, may help to explain the inability of immunodiscordant individuals to recover proper T_CM_/T_TM_ ratios.

### CD57 expression and immune recovery

**Figure 4:**
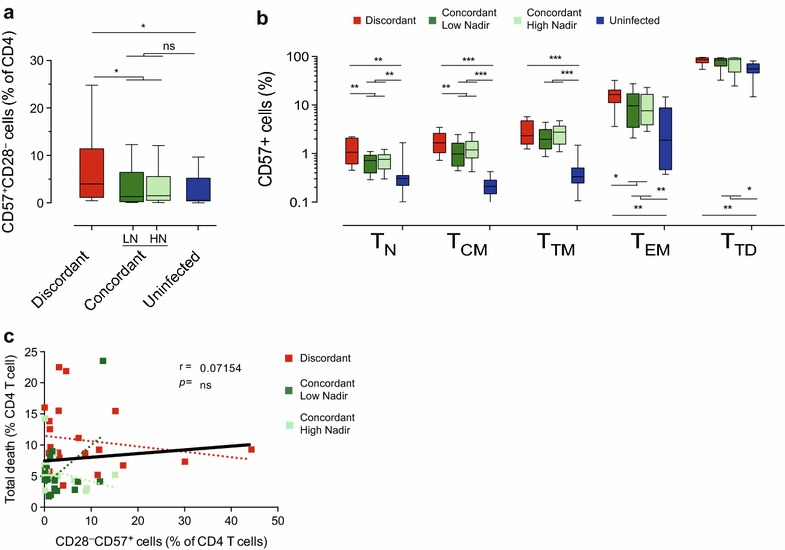
Characterization of CD57 expression in CD4 T cells. **a** The replicative senescence of the whole CD4 T-cell compartment was defined by the frequency of CD57^+^CD28^−^ cells. **b** In addition, the expression of CD57^+^ cells was evaluated in the different CD4 T cell subsets by analyzing the frequency of CD57^+^ cells. **c** The relationship between levels of spontaneous CD4 T-cell death and replicative immunosenescence is shown. Plots shows data from immunodiscordant (n = 23, *red*), low nadir immunoconcordant (n = 17, *dark green*) or high nadir immunoconcordant individuals (n = 16, *light green*) with color-coded linear regression for each data set. Linear regression for the global data is shown in *black lines*. Correlation coefficients and *p* *values* of Spearman’s test for the global analysis are shown. **a**, **b** also show data from HIV uninfected individuals (n = 11, *blue bars*).

Besides T_CM_ or T_TM_ dysfunction, replicative immunosenescence of CD4 T cells has also been related to immune recovery [14]. In our cohort, analysis of the frequency of CD28^−^CD57^+^ CD4 T cells (Additional file 1: Figure S1) showed higher levels in immunodiscordant subjects than in both immunoconcordant subgroups, which in turn showed similar levels to HIV-uninfected individuals (Figure 4a). The analysis of CD57 expression in the different CD4 T-cell subsets showed that despite apparent full immune recovery, immunoconcordant individuals displayed higher CD57 expression than uninfected controls in all CD4 T-cell subsets. In addition, immunodiscordant individuals showed highly significant differences with concordant subjects particularly in T_N_ and T_CM_ cells (Figure 4b).

We also evaluated the relationship of CD57 expression with CD4 T-cell survival. No significant correlation was found between replicative senescence (CD28^−^CD57^+^ cells) and spontaneous CD4 T-cell death (Figure 4c). These data suggest that different mechanisms regulate the expression of CD57 in different CD4 T-cell subsets and show that replicative senescence is not coupled with the spontaneous CD4 T-cell death rate characteristics of immunodiscordant responses to HAART.

### Analysis of the CD8 T-cell compartment

**Figure 5:**
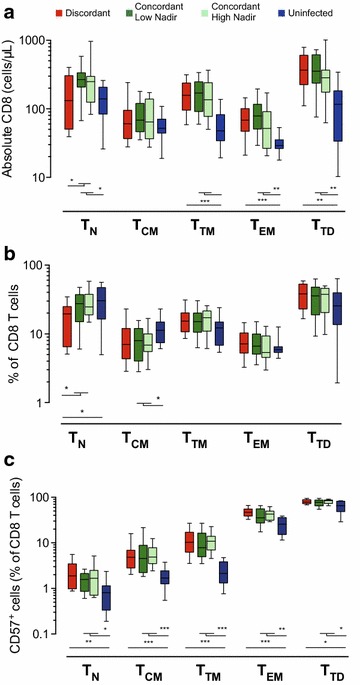
Characterization of CD8 T-cell subsets. The absolute number of circulating and T_N_, T_CM_, T_TM_, T_EM_ and T_TD_ CD8 T cells (**a**) was analyzed in immunodiscordant individuals (n = 23, *red boxes*), immunoconcordant individuals with low or high nadir (n = 17 and n = 16, *dark* and *light green boxes*, respectively) and healthy HIV uninfected individuals (n = 11, *blue boxes*). The frequency of the above-mentioned subsets in the CD8 T cell compartment (**b**) was also analyzed. The expression of CD57^+^ cells was further evaluated in the different subsets by the frequency of CD57^+^ cells (**c**). In all panels data shown correspond to median values (*bands*), IQR (*boxes*) and 10–90 interquartile values (*whiskers*) for immunodiscordant individuals (*red boxes*), immunoconcordant individuals with low or high nadir (*green boxes*) and healthy HIV uninfected individuals (*blue boxes*). Figure show *p* *values* (permutation test adjusted by false discovery rate).

Major comorbidities in HIV infected individuals have been related to CD8 T-cell activation [1, 22] and recently to the CD4/CD8 T-cell ratio. Therefore, we also evaluated differentiation and immunosenescence in CD8 T cells. Elevated CD8 T-cell counts in HIV infected individuals analyzed in this study (Table 1) were the consequence of an expansion of all subsets except CD8 T_CM_ cells (Figure 5a). Comparison of immunodiscordant and immunoconcordant subjects exclusively showed differences in the TN subset, consistent with the widely described thymic dysfunction of these patients [6]. The percentage of CD8 T cells was not normalized in immunoconcordant individuals, which showed intermediate values between immunodiscordant and uninfected subjects (Table 1). Among immunoconcordant and immunodiscordant individuals, T_TM_, T_EM_ and T_TD_ CD8 T-cell subsets showed similar frequencies. However, a decreased size of the CD8 T_N_ subset was observed in immunodiscordant individuals, while a reduced TCM subset was only observed in immunoconcordant subjects (Figure 5b). Replicative immunosenescence (CD28^−^CD57^+^ cells) was increased in both groups of HIV infected individuals compared to uninfected controls; however, no differences between immunoconcordant and immunodiscordant individuals were observed. A deeper analysis of CD57 expression in the different CD8 T-cell subsets commonly showed a lower expression of CD57 in uninfected donors compared to HIV infected groups, with no differences between immunodiscordant and immunoconcordant individuals (Figure 5c). Again, we observed similar CD57 expression when this latter group was subdivided according to nadir values of CD4 T-cell counts. In summary, individuals displaying different CD4 T-cell recovery profiles seem to have similar CD8 T-cell compartments, except for the number of CD8 T_N_ cells.

## Discussion

Immunodiscordant responses to HAART are associated with high risk of mortality and the occurrence of AIDS and non AIDS-related diseases [23–28]. Although blunted CD4 T-cell recovery has been associated with impaired thymic output [29], other factors affecting memory cell populations, such as activation, replicative immunosenescence [30], skewed T-cell maturation [15, 30, 31] and increased rates of CD4 T-cell death [8, 17], may contribute to persistently low CD4 T-cell counts.

The pivotal role of TN cells in CD4 T-cell repopulation and the lower size of this subset in immunodiscordant individuals has been universally reported [5, 10, 32, 33] and has been confirmed in our cohort using the CD31 marker (CD45RA^+^CD31^+^ percentage of CD4 T cells) [17]. However, the thymus hypothesis should not overlook the role of memory cell populations, which may maintain T-cell homeostasis in thymectomyzed SIV-infected animals [11]. Immunodiscordant individuals show low proliferation of T_N_ cells [15], suggesting that T_N_ cells might be not required for CD4 T-cell repopulation (Additional file 3: Figure S2). In the absence of new CD4 TN cells, memory cells should be expanded by homeostatic mechanisms involving IL-7 or TCR stimulation to maintain CD4 T-cell numbers [34, 35]. Since CD4 T_CM_ cells are the main target of IL-7 [36] and IL-7 levels are increased in our cohort of immunodiscordant individuals [37], the inability to recover CD4 T cells, could also be related to the limited expansion capacity of memory cells. It could be argued that the CD4 TCM cell subset is under strong homeostatic pressure. Supporting this notion, high TCM proliferation have been reported in immunodiscordant individuals [15]. However, the limited size of this subset in immunodiscordant patients indicates that the proliferation of CD4 T_CM_ cells may be inefficient or may result in increased maturation towards T_TM_ or T_EM_ phenotypes. This could explain the relative accumulation of CD4 T cells in intermediate stages of differentiation, mainly TTM but also TEM cells, observed in our cohort of immunodiscordant individuals. Interestingly, our data show an association between the levels of CD4 T_TM_ cells and the rate of spontaneous CD4 T-cell death, although the analysis of cell death in sorted CD4 T-cell subsets in immunodiscordant and immunoconcordant individuals indicates that CD4 T_CM_ cell death is better correlated to CD4 T-cell recovery. Our data suggest that reduced T_CM_ survival may be a second mechanism that adds to low thymic output in immunodiscordant patients.

An additional consequence of the high cell death level of TCM and the trends detected in T_TM_ or T_EM_ cells in immunodiscordant patients is the modest accumulation of senescent CD4 T cells. Since the fate of CD4 T_CM_ and T_TM_ or T_EM_ could be cell death rather than further maturation (Additional file 3: Figure S2), this fact could explain the poor relationship between immunodiscordance and replicative senescence. Regarding senescence, our data on CD57 expression are consistent with previous reports [29], revealing higher levels of CD28^–^CD57^+^CD4 T cells in immunodiscordant individuals. Although some reports suggest that CD57^+^ CD4 T-cells undergo faster spontaneous apoptosis [38], the frequency of these cells is not associated with cell death rate in the CD4 T-cell compartment, suggesting that they are unlikely to drive increased cell death in immunodiscordant individuals. Consistently, several authors suggest that CD28^−^CD57^+^ cells are long lived [39, 40]. Aside from replicative senescence, the expression of CD57 in other CD4 T-cell subsets, namely CD4 T_N_ cells, has been also associated with CD4 T-cell recovery [30]. Although we have also noticed this association, it remains unclear whether CD57^+^ cells in T_N_ and T_CM_ subsets are actually senescent [12], or reflect other T-cell functions or subsets such as Follicular helper CD4 T-cells (T_Fh_), which express CD57 [41] and are recovered after HAART [42]. The increased expression of CD57 in CD4 T_N_ and T_CM_ cells from immunodiscordant individuals points to alterations in these subsets and suggests again a role for T_CM_ cells in CD4 T-cell recovery.

Our data point out that the level of destruction of the immune system, as measured by the nadir CD4 T-cell count, which has been widely associated with poor CD4 T-cell recovery [5], fail to fully identify immunodiscordant responses. Indeed, some individuals with low nadir values are able to recover the CD4 T-cell compartment to the same extent as subjects with higher nadir values, at least in respect to maturation phenotypes. Although this observation does not occlude the fact that starting HAART with low CD4 T-cell counts increases the risk of immunodiscordant responses, it suggests that not only the number but also the quality of CD4 T cells determine immune reconstitution. In this regard, it is tempting to speculate that the size of the TCM compartment prior to HAART may yield a better predictive value [16].

We have also shown that despite the impairment/failure of thymic output in both CD4 and CD8 T-cell subsets, only CD4 T cells show an unbalanced maturation profile. While minimal differences were observed among HIV infected groups in CD8 T cells. This result is surprising since CD8 T-cell senescence or activation has also been related to clinical progression in treated HIV infected individuals [43, 44]. Moreover, a recent analysis of HIV infected individuals failing to recover CD4/CD8 T-cell ratios after HAART also points to maturation and activation of CD8 T cells as major markers of poor immune recovery [45]. This apparent paradox could be related to the different pressures acting on CD4 and CD8 T cells or to the specific features of our cohort, which exclusively included long-term treated individuals (more than 2 years) without AIDS defining events. It remains possible that we have selected individuals with low risk of clinical progression, potentially minimizing differences in CD8 T cells. On the other hand, our selection criteria and classification cutoff, based exclusively in CD4 T-cell counts, allows for the direct comparison of the CD4 T-cell compartment between groups, avoiding the confounding effects of inflammatory and clinical status. In contrast, our analysis has some limitations. First, inclusion criteria might select immunodiscordant patients with a trend towards a lower treatment time (see Table 1). However, correcting maturation and senescence analyses for time on treatment did not modify the main conclusions of the study (not shown). A second limitation comes from the definition of immunodiscordant responses. We have used a cutoff value of 350 CD4 T cells/µL, while other authors define the limit in higher values. To assess the robustness of our analysis, a cutoff of 500 CD4 T cells/µL has been also used, yielding similar results (data not shown). Finally, another confounding factor relevant in the context of this study is CMV co-infection, a major driver of immunosenescence. However, all HIV infected individuals and 9 out of 11 uninfected individuals recruited were seropositive for CMV infection. Exclusion of CMV seronegative controls did not modify our analysis.

## Conclusions

In summary, our data point to a major perturbation of the central memory CD4 T cells as a new prominent characteristic of immunodiscordant responses to HAART. This perturbation adds to the increased accumulation of senescent cells and to the poor thymic output, and points to CD4 T_CM_ and T_TM_ cells as the major players of CD4 T-cell repopulation.

## Additional files

Additional file 1:
**Figure S1.** Gating strategy followed to identify T-cell maturation stages. **Panel A.** CD4 and CD8 T cells were gated and analyzed for the expression of CD27 and CD28. For CD4 T cells, terminally differentiated (T_TD_) cells were defined as CD28–CD27^−^, while effector memory (T_EM_) cells were CD28^+^CD27^−^. Double positive cells were further analyzed for CCR7 and CD45RA expression to identify naïve (T_N_) cells (CCR7^+^CD45RA^+^), central memory (T_CM_) cells (CCR7^+^CD45RA^−^) and transitional Memory (T_TM_) cells (CCR7^–^CD45RA^−^). **Panel B**. For CD8 T cells, the general strategy was similar, unless for the definition of T_EM_ cells that was CD27^+^CD28^–^ cells. The expression of CD57 was analyzed in the whole CD4 or CD8 T-cell population (lower left dot plot in each panel) to define replicative senescence (CD28^–^CD57^+^ cells). In addition the expression of CD57 in each subset was also evaluated (lower dot plots in each panels). Additional file 2:
**Table S1.** **A**. The multivariate relationship between T_N_, T_CM_, T_TM_, T_EM_, T_TD_ and Total Death were determined by multiple linear regression. **B**. Linear regression model shows no statistical differences between group of patients (Discordant/Concordant) in the association of Total Death with T_TM_. Additional file 3:
**Figure S2.** A model for CD4 homeostasis in Immunodiscordant individuals. Immunoconcordant individuals show a full recovery of CD4 T-cell counts with a high representation of naïve cells, and balanced frequencies of different memory subsets (upper panels). The profile of CD4 T-cell maturation in immunoconcordant individuals (green line) overlaps with that of HIV uninfected individuals (blue line in upper right plot) in which CCR7^−^ CD4 T cells show increased turnover and short live [22]. Conversely,immunodiscordant individuals (lower panels) show a reduced naïve subset with no signs of altered turnover [14] that limits the generation of new memory cells. Among memory subsets, central memory cells are also reduced and subjected to homeostatic pressure to generate new cells [14] increasing T_CM_→T_TM_ transition (and lowering T_CM_/T_TM_ ratios) and increasing T_CM_ cell death. T_TM_ and T_EM_ cells emerging from T_CM_ cells also show higher sensitivity to cell death (Figure 2D), explaining the lack of accumulation of terminally differentiated cells in these subjects. This scenario results in a shifted profile of CD4 T-cell maturation (red line) compared to healthy individuals (blue line in lower right plot).
