# Supplementary material for: Increased ex vivo cell death of central memory CD4 T cells in treated HIV infected individuals with unsatisfactory immune recovery
Source: J Transl Med. 2015 Jul 17;13:230. doi: 10.1186/s12967-015-0601-2 (PMC4504072; doi:10.1186/s12967-015-0601-2)
Supplement: Additional file 1: — Figure S1. Gating strategy followed to identify T-cell maturation stages. Panel A. CD4 and CD8 T cells were gated and analyzed for the expression of CD27 and CD28. For CD4 T cells, terminally differentiated (TTD) cells were defined as CD28–CD27−, while effector memory (TEM) cells were CD28+CD27−. Double positive cells were further analyzed for CCR7 and CD45RA expression to identify naïve (TN) cells (CCR7+CD45RA+), central memory (TCM) cells (CCR7+CD45RA−) and transitional Memory (TTM) cells (CCR7–CD45RA−). Panel B. For CD8 T cells, the general strategy was similar, unless for the definition of TEM cells that was CD27+CD28– cells. The expression of CD57 was analyzed in the whole CD4 or CD8 T-cell population (lower left dot plot in each panel) to define replicative senescence (CD28–CD57+ cells). In addition the expression of CD57 in each subset was also evaluated (lower dot plots in each panels). [file 12967_2015_601_MOESM1_ESM.pdf]

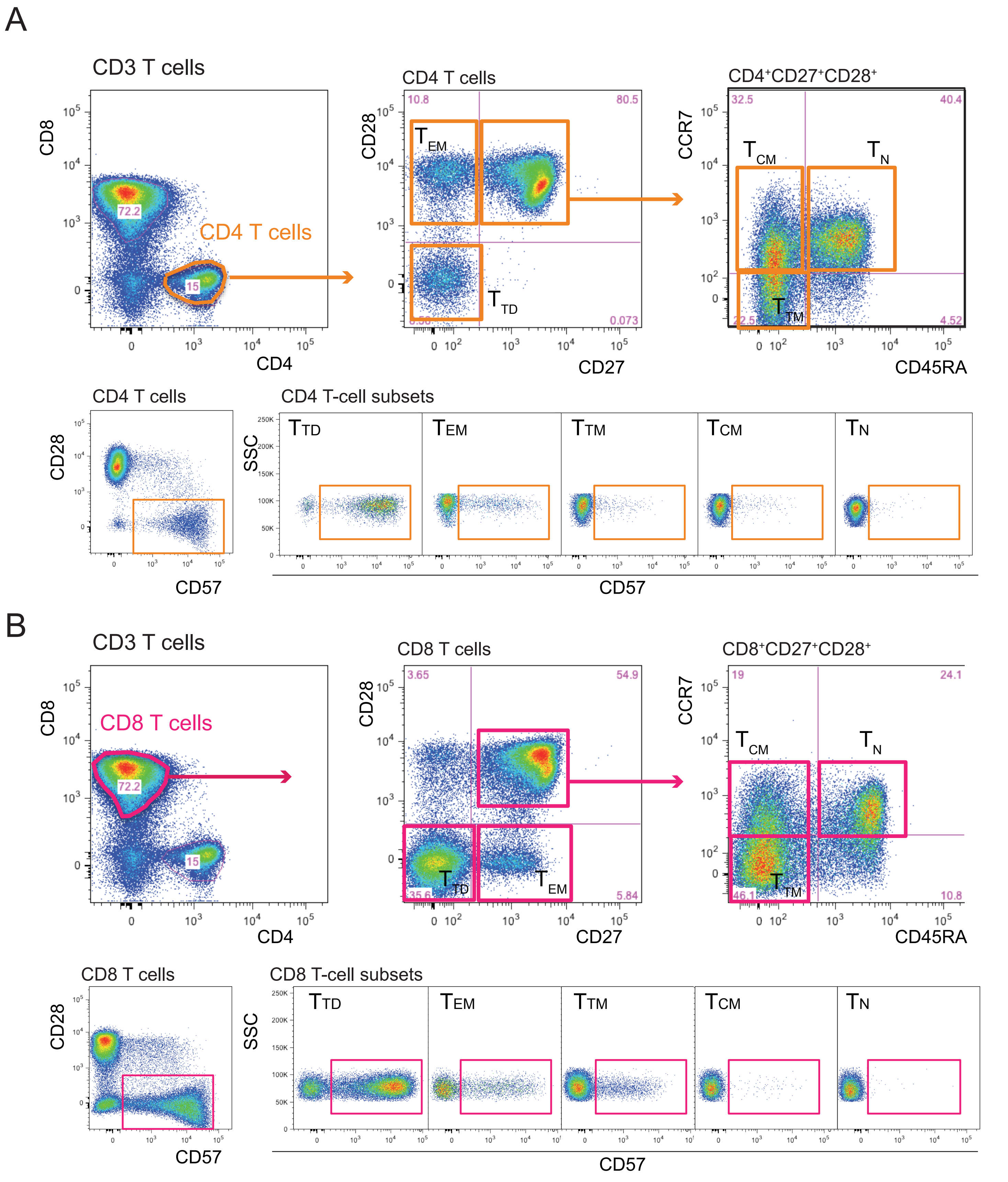

Supplementary Figure 1. Gating strategy followed to identify T-cell maturation stages. **Panel A.** CD4 and CD8 T cells were gated and analyzed for the expression of CD27 and CD28. For CD4 T cells, terminally differentiated (T_{TD}) cells were defined as $CD28^{-}CD27^{-}$, while effector memory (T_{EM}) cells were $CD28^{+}CD27^{-}$. Double positive cells were further analyzed for CCR7 and CD45RA expression to identify naïve (T_N) cells ($CCR7^{+}CD45RA^{+}$), central memory (T_{CM}) cells ($CCR7^{+}CD45RA^{-}$) and transitional Memory (T_{TM}) cells ($CCR7^{-}CD45RA^{-}$). **Panel B.** For CD8 T cells, the general strategy was similar, unless for the definition of T_{EM} cells that was $CD27^{+}CD28^{-}$ cells. The expression of CD57 was analyzed in the whole CD4 or CD8 T-cell population (lower left dot plot in each panel) to define replicative senescence ($CD28^{-}CD57^{+}$ cells). In addition the expression of CD57 in each subset was also evaluated (lower dot plots in each panels).
