# Supplementary material for: Increased ex vivo cell death of central memory CD4 T cells in treated HIV infected individuals with unsatisfactory immune recovery
Source: J Transl Med. 2015 Jul 17;13:230. doi: 10.1186/s12967-015-0601-2 (PMC4504072; doi:10.1186/s12967-015-0601-2)
Supplement: Additional file 2: — Table S1. A. The multivariate relationship between TN, TCM, TTM, TEM, TTD and Total Death were determined by multiple linear regression. B. Linear regression model shows no statistical differences between group of patients (Discordant/Concordant) in the association of Total Death with TTM. [file 12967_2015_601_MOESM2_ESM.pdf]

Supplementary Table 1

Table S1.A. The multivariate relationship between T_{N}, T_{CM}, T_{TM}, T_{EM}, T_{TD} and Total Death were determined by multiple linear regression

Full Model [a]					Final Model [b]				
	Estimate	Std. Error	t value	Pr(>|t|)		Estimate	Std. Error	t value	Pr(>|t|)
(Intercept)	13.8103	4.5829	3.01	0.0043	(Intercept)	13.2695	4.1680	3.18	0.0026
Naive	0.0009	0.0161	0.06	0.9534	Transitionals	0.1791	0.0349	5.13	0.0000
Central Memory	0.0290	0.0544	0.53	0.5962					
Transitionals	0.1179	0.0817	1.44	0.1560					
Effector	0.0116	0.0356	0.33	0.7467					
Term diff	0.0142	0.0549	0.26	0.7976					

[a]Model with all predictor variables. [b]Final model (adj R^2=0.34, *P*-value <0.001) derived from Stepvise procedure using the akaike information criterion (AIC).

Table S1.B. Linear regression model shows no statistical differences between group of patients (Discordant/Concordant) in the association of Total Death with T_{TM}.

	Estimate	Std. Error	t value	Pr(>|t|)
(Intercept)	13.1098	5.5317	2.37	0.0220
Transitionals	0.1799	0.0399	4.51	0.0000
Transitionals:Discordant	0.0027	0.0603	0.04	0.9647
