# Supplementary material for: Increased ex vivo cell death of central memory CD4 T cells in treated HIV infected individuals with unsatisfactory immune recovery
Source: J Transl Med. 2015 Jul 17;13:230. doi: 10.1186/s12967-015-0601-2 (PMC4504072; doi:10.1186/s12967-015-0601-2)
Supplement: Additional file 3: — Figure S2. A model for CD4 homeostasis in Immunodiscordant individuals. Immunoconcordant individuals show a full recovery of CD4 T-cell counts with a high representation of naïve cells, and balanced frequencies of different memory subsets (upper panels). The profile of CD4 T-cell maturation in immunoconcordant individuals (green line) overlaps with that of HIV uninfected individuals (blue line in upper right plot) in which CCR7− CD4 T cells show increased turnover and short live [22]. Conversely,immunodiscordant individuals (lower panels) show a reduced naïve subset with no signs of altered turnover [14] that limits the generation of new memory cells. Among memory subsets, central memory cells are also reduced and subjected to homeostatic pressure to generate new cells [14] increasing TCM→TTM transition (and lowering TCM/TTM ratios) and increasing TCM cell death. TTM and TEM cells emerging from TCM cells also show higher sensitivity to cell death (Figure 2D), explaining the lack of accumulation of terminally differentiated cells in these subjects. This scenario results in a shifted profile of CD4 T-cell maturation (red line) compared to healthy individuals (blue line in lower right plot). [file 12967_2015_601_MOESM3_ESM.pdf]

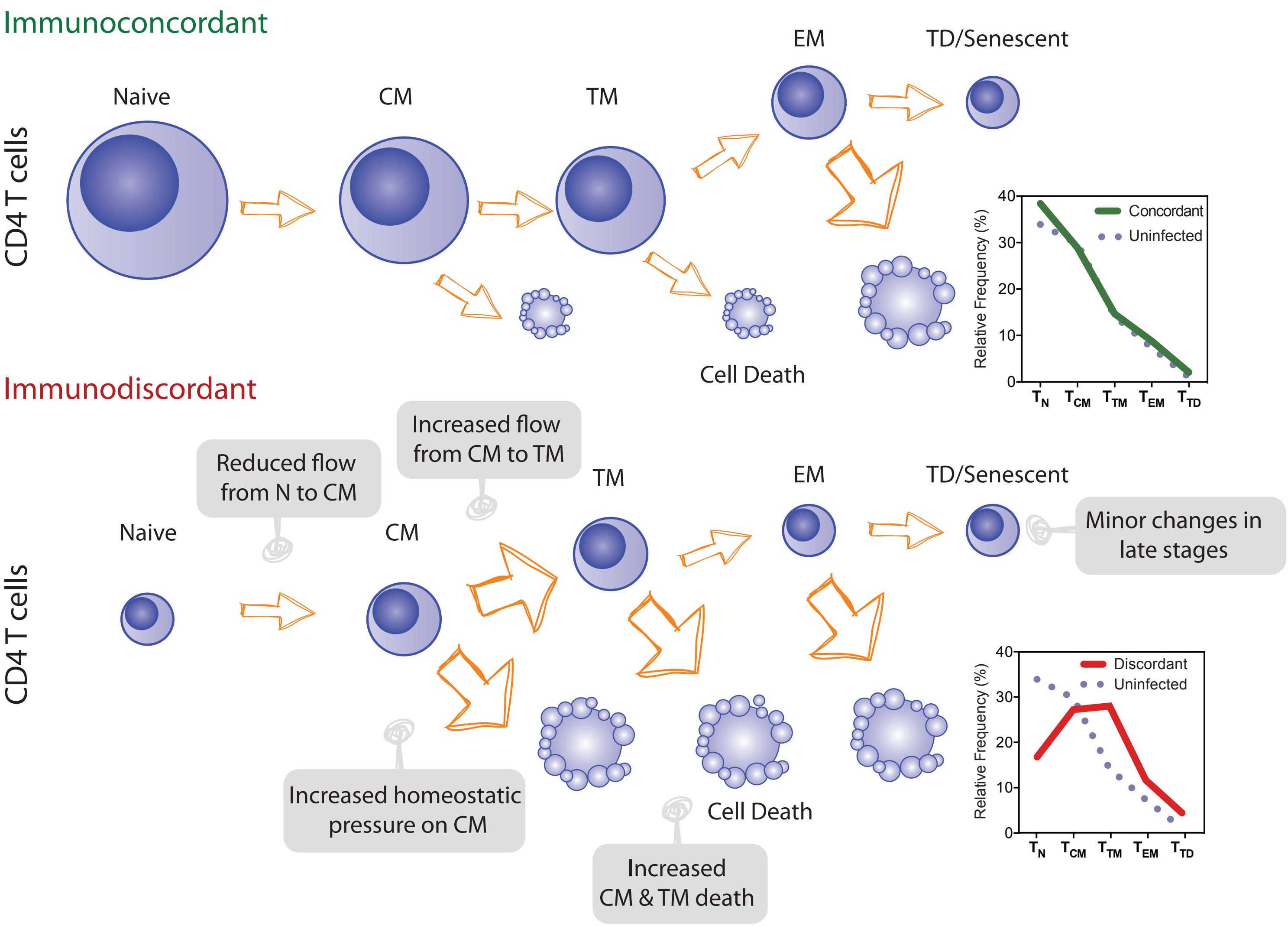

Supplementary Figure 2. A model for CD4 homeostasis in Immunodiscordant individuals. Immunoconcordant individuals show a full recovery of CD4 T-cell counts with a high representation of naïve cells, and balanced frequencies of different memory subsets (upper panels). The profile of CD4 T-cell maturation in immunoconcordant individuals (green line) overlaps with that of HIV uninfected individuals (blue line in upper right plot) in which $CCR7^{-}$ CD4 T cells show increased turnover and short live [22]. Conversely,immunodiscordant individuals (lower panels) show a reduced naïve subset with no signs of altered turnover [14] that limits the generation of new memory cells. Among memory subsets, central memory cells are also reduced and subjected to homeostatic pressure to generate new cells [14] increasing $T_{CM} \rightarrow T_{TM}$ transition (and lowering T_{CM}/T_{TM} ratios) and increasing T_{CM} cell death. T_{TM} and T_{EM} cells emerging from T_{CM} cells also show higher sensitivity to cell death (Figure 2D), explaining the lack of accumulation of terminally differentiated cells in these subjects. This scenario results in a shifted profile of CD4 T-cell maturation (red line) compared to healthy individuals (blue line in lower right plot).
